# Methylation of *BRCA1* and *MGMT* genes in white blood cells are transmitted from mothers to daughters

**DOI:** 10.1186/s13148-018-0529-5

**Published:** 2018-07-26

**Authors:** Nisreen Al-Moghrabi, Maram Al-Showimi, Nujoud Al-Yousef, Bushra Al-Shahrani, Bedri Karakas, Lamyaa Alghofaili, Hannah Almubarak, Safia Madkhali, Hind Al Humaidan

**Affiliations:** 1 Head of Cancer Epigenetic Section, Molecular Oncology Department, King Faisal Specialist Hospital and Research Centre, PO BOX 3354, Riyadh, 11211 Kingdom of Saudi Arabia; 2 Cancer Epigenetic section, Department of Molecular Oncology, King Faisal Specialist Hospital and Research Centre, PO BOX 3354, Riyadh, 11211 Kingdom of Saudi Arabia; 3 Al Faisal University College of Medicine, PO BOX 50927, Riyadh, 11533 Kingdom of Saudi Arabia; 4 King Saud bin Abdulaziz University for Health Sciences, PO BOX 22490, Riyadh, 3130 Kingdom of Saudi Arabia; 5 Department of pathology and Laboratory Medicine, King Faisal Specialist Hospital and Research Centre, PO BOX 3354, Riyadh, 11211 Kingdom of Saudi Arabia

**Keywords:** *BRCA1*, *MGMT*, Methylation, Transmission, Blood, Breast cancer, Ovarian cancer

## Abstract

**Background:**

Constitutive methylation of tumor suppressor genes are associated with increased cancer risk. However, to date, the question of epimutational transmission of these genes remains unresolved. Here, we studied the potential transmission of *BRCA1* and *MGMT* promoter methylations in mother-newborn pairs.

**Methods:**

A total of 1014 female subjects (cancer-free women, *n* = 268; delivering women, *n* = 295; newborn females, *n* = 302; breast cancer patients, *n* = 67; ovarian cancer patients, *n* = 82) were screened for methylation status in white blood cells (WBC) using methylation-specific PCR and bisulfite pyrosequencing assays. In addition, *BRCA1* gene expression levels were analyzed by quantitative real-time PCR.

**Results:**

We found similar methylation frequencies in newborn and adults for both *BRCA1* (9.9 and 9.3%) and *MGMT* (12.3 and 13.1%). Of the 290 mother-newborn pairs analyzed for promoter methylation, 20 mothers were found to be positive for *BRCA1* and 29 for *MGMT*. Four mother-newborn pairs were positive for methylated *BRCA1* (20%) and nine pairs were positive for methylated *MGMT* (31%). Intriguingly, the delivering women had 26% lower *BRCA1* and *MGMT* methylation frequencies than those of the cancer-free female subjects. *BRCA1* was downregulated in both cancer-free woman carriers and breast cancer patients but not in newborn carriers. There was a statistically significant association between the *MGMT* promoter methylation and late-onset breast cancers.

**Conclusions:**

Our study demonstrates that *BRCA1*and *MGMT* epimutations are present from the early life of the carriers. We show the transmission of *BRCA1* and *MGMT* epimutations from mother to daughter. Our data also point at the possible demethylation of *BRCA1*and *MGMT* during pregnancy.

## Background

Defects in epigenetic manipulation, which results in the atypical transcriptional silencing of active genes and/or reactivation of silent genes, are defined as “Epimutation” [1]. This non-genetic change is a potent mechanism responsible for the suppression of various tumor suppressor genes; hence, it is considered as a mechanism for cancer predisposition [2]. The presence of epimutation in all animal tissues could be either germ line, with evidence of inheritance, or constitutional, with no evidence of inheritance [3–5]. DNA repair genes have been reported to be inactivated in many cancer types by epigenetic silencing mechanism. Deficiencies in these genes usually lead to genetic instability, which is an important mechanism in cancer initiation and/or progression.

*BRCA1* is a DNA repair gene that is expressed in all mammalian cells. This gene plays an important role in the error-free pathway of homologous recombination [6], which repairs double-strand breaks. Cells that lack BRCA1 protein are prone to acquire mutations and chromosomal rearrangements, which can lead to carcinogenesis. It is well established that germline *BRCA1* mutations are responsible for many familial cancer types including breast and ovarian cancers [7]. Similarly, methylation in the *BRCA1* promoter is a mechanism for *BRCA1* inactivation during early carcinogenesis. Constitutive *BRCA1* methylation has been found to be associated with a 3.5-fold increase in the risk of developing early-onset breast cancer and a major predisposition factor for serous ovarian cancer [8–13]. This renders the constitutive *BRCA1* promoter methylation as a potential predictive biomarker for breast and ovarian cancer predisposition [12].

*MGMT* is another DNA repair gene that is also inactivated in human cancers by promoter methylation [14, 15]. It is involved in the removal of an alkyl group from the O^6^ position of the guanine nucleotide [16]. The loss of *MGMT* activity leads to G>A transition due to the inability of removing the mutagenic adducts from guanine [17] resulting in DNA aberrations and tumor progression [18]. It has been reported that *MGMT* methylation is a common mechanism in triple negative breast cancers (TNBC) where it has been detected in 83.1% of the cases with a weak association with advanced age [19]. Furthermore, *MGMT* promoter methylation and the lack of *MGMT* expression were found to be associated with the mucinous and clear cell subtypes of epithelial ovarian cancer [20]. To date, the prevalence of *MGMT* methylation in cancer-free individuals and its potential inheritance have not been studied.

Transgenerational epigenetic inheritance is the passage of epigenetic markers, such as DNA methylation, through germline from one generation to the next. Evidences of epimutation inheritance have been reported for the DNA mismatch repair genes *MLH1* and *MSH2* [21–23]. Since no association has been found between the presence of *BRCA1* methylation in peripheral blood cells and age [9, 10], it has been also suggested that *BRCA1* epimutation might be inherited. However, up to date, the question of germ line *BRCA1*epimutation inheritance remains unresolved.

In this study, we investigated the prevalence of *BRCA1* and *MGMT* promoter methylations in white blood cells (WBC) from cancer-free women and newborn females. In addition, we investigated the potential transmission of the epimutation of the two genes from mother to daughter in mother-newborn female pairs.

## Results

### Cancer-free women and newborns have similar frequencies of WBC *BRCA1* promoter methylation

**Fig. 1:**
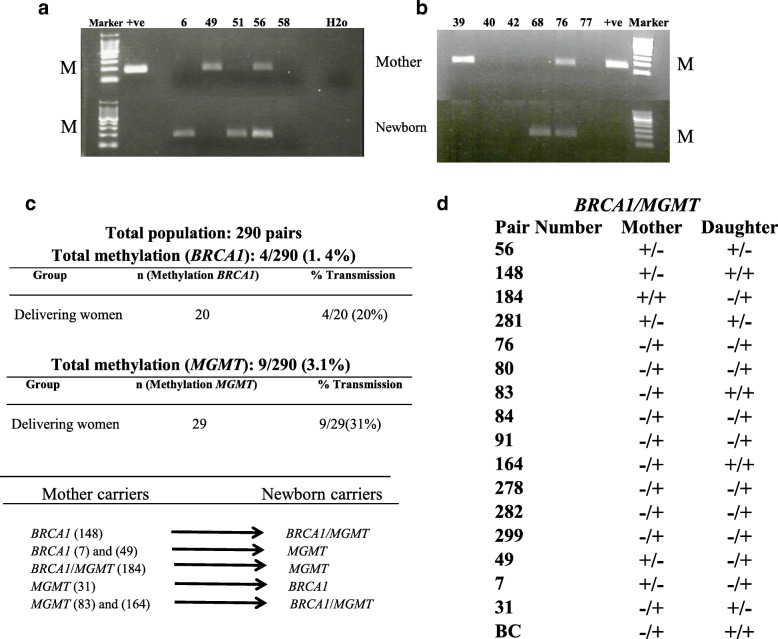
*BRCA1*and *MGMT* promoter methylation status in DNA from WBC. MSP analysis of **a** *BRCA1* promoter and **b** *MGMT* promoter. Totally methylated bisulfite-modified DNA was used as positive (+ve) control. Only the methylated bands are shown (M). Top panel for mothers, bottom panel for newborns. **c**, **d** Summary of *BRCA1* and *MGMT* methylation transmission from mothers to daughters. (+) positive for methylation, (−) negative for methylation

To investigate the potential transmission of methylated *BRCA1* promoter from mother to daughter, we examined the *BRCA1* promoter methylation status in DNA from WBC using MSP assay in a cohort of 865 female subjects (cancer-free women, *n* = 268; delivering women, *n* = 295; newborn females, *n* = 302). The cohort of the mothers and newborns included 290 mother-newborn pairs. We detected the *BRCA1* promoter methylation in 25 of 268 (9.3%) cancer-free women and in 20 of 295 (6.8%) delivering women (Fig. 1a and Table 1). Interestingly, 30 of 302 (9.9%) newborns were positive for the methylated *BRCA1* promoter. This shows that cancer-free women and newborns have similar frequencies of *BRCA1* promoter methylation in their WBC.

**Table 1:** Percentage of WBC DNA *BRCA1* and *MGMT* methylations

	Total population (*n* = 1014)	
Gene	Group	Promoter methylation (%)
*BRCA1*	Control women	25/268 (9.3)
	Delivering women	20/295 (6.8)
	Newborns	30/302 (9.9)
	Breast cancer	5/67 (7.5)
	Ovarian cancer	13/82 (15.8)
*MGMT*	Control women	35/268 (13.1)
	Delivering women	29/295 (9.8)
	Newborns	37/302 (12.3)
	Breast cancer	10/67 (15)
	Ovarian cancer	17/82 (20.7)
	Group	Methylation (%)
*BRCA1/MGMT*	Control women	6/25 (24)
	Delivering women	2/20 (10)
	Newborns	3/30 (10)
	Breast cancer	0
	Ovarian cancer	5/17 (29.4)

### Cancer-free woman and newborn carriers have similar levels and pattern of WBC *BRCA1* promoter CpG Island methylation

**Fig. 2:**
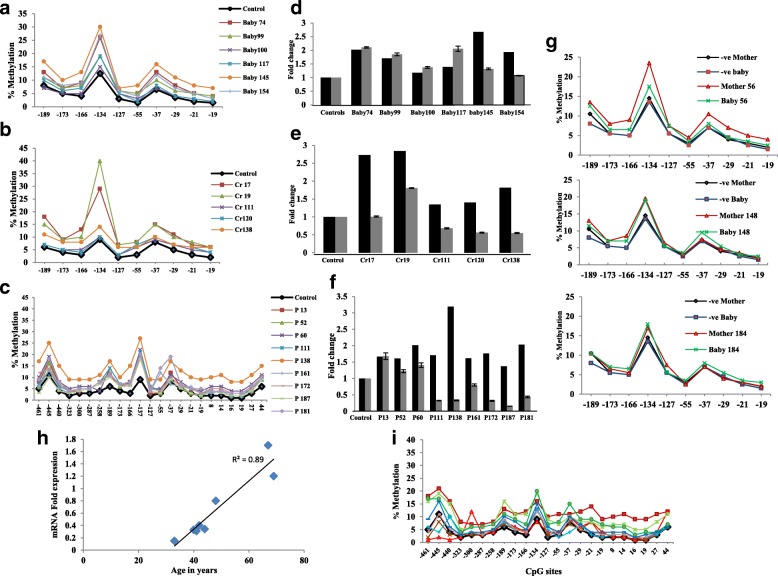
Methylation plots for *BRCA1* promoter measured by bisulfite pyrosequencing assay. Levels and pattern of methylation of CpG sites along the *BRCA1* promoter region in WBC from **a** newborn carriers, **b** woman carriers, **c** breast cancer patients, and **i** ovarian cancer patients. **g** Methylation plots for mother-newborn pairs. Black lines represent average values for control unmethylated samples, and colored lines represent single individuals. Numbers represent CpG sites relative to transcription start site. **d**–**f** Effect of promoter methylation on *BRCA1* mRNA expression in WBC from newborn carriers, woman carriers, and breast cancer patients, respectively. Black bars represent fold change in *BRCA1* promoter methylation. Gray bars represent fold change in *BRCA1* expression. Cr carrier, P patient. **h** Correlation between *BRCA1* mRNA levels in WBC and patient age. *R*^2^ correlation coefficient

To further elucidate the *BRCA1* promoter methylation status in newborn carriers as compared to woman carriers, we analyzed the level and the pattern of the *BRCA1* promoter methylation in their WBC. The methylation levels and patterns were studied by sodium bisulfite pyrosequencing in 10 CpG sites located in the *BRCA1* promoter at the 5′ flanking region. This region is known to have a strong promoter activity. Both women and newborns’ WBC DNA showed a distinct pattern of *BRCA1* methylation wherein − 134 and − 37 sites showed higher levels of methylation compared to other sites (Fig. 2a, b). Furthermore, both DNA types contained similar levels of methylation across the 10 CpG sites. This indicates that the level and pattern of WBC *BRCA1* promoter methylation are similar in woman and newborn carriers.

### *BRCA1* epimutation is transmitted from mother to daughter

Interestingly, we found four out of the 20 mothers (20%), who were tested positive for *BRCA1* methylation, had *BRCA1* methylation-positive daughters (Fig. 1c, d). This result is the first indication of the transmission of *BRCA1* epimutation from mother to daughter. To further verify the methylation in the positive mother-newborn pairs, the promoter region was analyzed by pyrosequencing in three pairs (Fig. 2g). Importantly, both mothers and newborns’ WBC DNA showed similar pattern and levels of methylation across the CpG sites analyzed. Importantly, we found one of the newborn carriers, who have a *BRCA1* methylation-negative mother, has also a *BRCA1* methylation negative father.

### *MGMT* promoter is methylated in both cancer-free women and newborns

We have previously shown that the *MGMT* gene is methylated in WBC of cancer-free *BRCA1* methylation carriers [24]. Thus, in this study, we sought to investigate whether there is an association between the presence of *BRCA1* and *MGMT* promoter methylations in WBC. To this end, we analyzed the *MGMT* promoter methylation in WBC using MSP assay in the same cohort of 865 cancer-free females. We detected the *MGMT* methylation in 35 of 268 (13.1%) cancer-free women, in 29 of 295 (9.8%) delivering women, and in 37 of 302 (12.3%) newborns (Fig. 1b and Table 1). These results show a high prevalence of methylated *MGMT* promoter in both adult and newborns. Importantly, we found six women (24%), two delivering women (10%), and three newborns (10%) to be positive for paired *BRCA1*/*MGMT* methylation (Table 1).

### *MGMT* epimutation is transmitted from mother to daughter

Interestingly, nine out of the 29 mothers (31%), who were tested positive for *MGMT* methylation, had *MGMT* methylation-positive daughters (Fig. 1c, d). This is also the first reported result suggesting the transmission of *MGMT* epimutation from mother to daughter. Additionally interesting, we found two *BRCA1* methylation-positive mothers having *MGMT* methylation-positive daughters and vice versa (Fig. 1c, d). Notably, the mother of a *BRCA1* woman carrier was a breast cancer patient who was positive for methylated *MGMT* (Fig. 1d).

### *MGMT* promoter methylation is associated with ovarian cancer and the late onset of breast cancer

In order to value the epimutation of *MGMT* and *BRCA1* in WBC from cancer-free women and newborns, we investigated the prevalence of the methylated *BRCA1* and *MGMT* promoters in breast and ovarian cancer patients. To this end, we screened 67 breast and 82 ovarian cancer patients using MSP assay. We found that 5 out of 67 (7.5%) breast and 13 out of 82 (15.8%) ovarian cancer patients tested positive for *BRCA1* promoter methylation (Table 1). Moreover, 10 of 67 (15%) breast and 17 of 82 (20.7%) ovarian cancer patients were positive for *MGMT* methylation (Table 1). We did not detect any case with both *BRCA1* and *MGMT* methylations in breast cancer patients. However, in a cohort of 17 breast cancer patients who were tested positive for *BRCA1* methylation in our previous study [24], four patients (23.5%) were found to be positive for *MGMT* methylation (Table 2). Interestingly, we found that the mean age for the onset of breast cancer in the *BRCA1* methylation-positive patients was 40.3 ± 6.4 (95%CI 37.1–43.4) years compared to 50.9 ± 12.7 (95%CI 41.8–60) years for methylated *MGMT* and 56 ± 14.1(95%CI 33.8–78.7) years for both *BRCA1/MGMT-*methylated patients (*p* = 0.0044). This indicates a significant association between the *MGMT* methylation and late onset of the disease, (*p* = 0.0253) for *MGMT* alone and (*p* = 0.0157) for paired *BRCA1/MGMT*. Importantly, five of the 13 (38.5%) *BRCA1* methylation-positive ovarian cancer patients had methylated *MGMT* gene. However, no association was found between the *MGMT* methylation and the onset of the disease (Table 3).

**Table 2:**
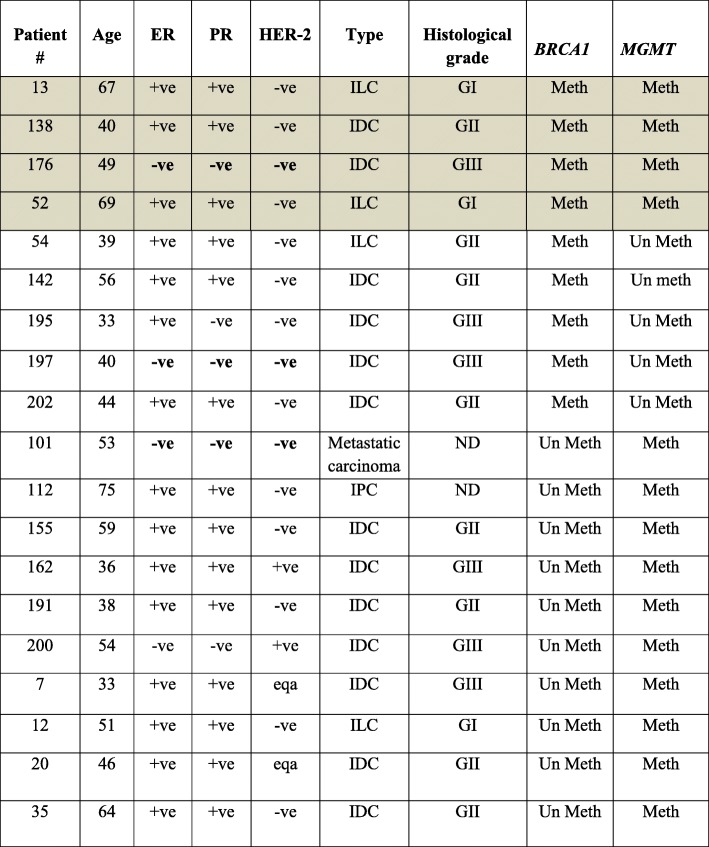
Clinical characterizations of *BRCA1*- and *MGMT*-methylated breast cancer-positive cases

Shaded area specifies patients identified in our previous study (reference [24])

*ILC* invasive lobular carcinoma, *IDC* invasive ductal carcinoma, *ND* no data

**Table 3:** Clinical characterizations of *BRCA1*- and *MGMT*-methylated ovarian cancer patients

Patient no.	Age	Type	Grade	*BRCA1*	*MGMT*
2	54	Clear cell carcinoma	Advanced	Meth	Meth
13	54	Serous carcinoma	High	Meth	Meth
36	55	Ovarian serous carcinoma	High	Meth	Meth
50	43	Ovarian serous carcinoma.	High	Meth	Meth
23	40	Serous carcinoma	High	Mut/Meth	Meth
38	54	Serous carcinoma	ND	Mut/Meth	Un Meth
7	57	Papillary serous carcinoma	High	Meth	Un Meth
24	53	Serous adenocarcinoma	3	Meth	Un Meth
27	47	Serous carcinoma involving uterus	High	Meth	Un Meth
52	67	ovarian adenocarcinoma	High	Meth	Un Meth
59	53	ovarian serous carcinoma	High	Meth	Un Meth
71	38	Ovarian serous carcinoma	ND	Meth	Un Meth
29	47	Carcinoma of the right ovary	High	Meth	Un Meth
6	58	Papillary serous carcinoma	High	Mut	Meth
14	34	Serous adenocarcinoma	High	Mut	Meth
47	65	Poorly differentiated adenocarcinoma	ND	Mut	Meth
17	66	serous ovarian carcinoma	ND	Mut	Meth
69	41	Ovarian serous carcinoma	ND	Mut	Meth
4	38	Clear cell carcinoma	GII	WT	Meth
9	46	papillary serous cancer	High	WT	Meth
16	67	Serous adenocarcinoma	High	WT	Meth
44	88	Metastatic granulosa cell tumor	ND	WT	Meth
55	43	serous carcinoma	High	WT	Meth
60	49	Granulosa cell tumor	ND	WT	Meth
79	44	Mucinous cyst adenocarcinoma	ND	WT	Meth

*Meth* methylated, *Mut* mutated, *WT* wild type, *ND* no data

### *BRCA1* expression is reduced in breast cancer patients and woman carriers but not in newborn carriers

Next, we sought to assess the expression of *BRCA1*, at the level of mRNA, in WBC. To this end, we analyzed the expression level of the *BRCA1* gene by real-time RT-PCR in the newborn carriers, woman carriers, and *BRCA1* methylation-positive breast cancer patients. Interestingly, we did not find any reduction in the expression level of the *BRCA1* in six highly methylated newborns as compared to unmethylated controls (Fig. 2a, d). However, in woman carriers, the expression level was reduced by two folds in three out of five woman carriers (Fig. 2b, e). Furthermore, we found a considerable reduction in the expression level of the *BRCA1* in six out of nine breast cancer patients (Fig. 2c, f). Interestingly, the fold change of the *BRCA1* expression level in breast cancer patients highly correlated (*R* = 0.89) with patient’s age in eight out of nine cases (Fig. 2h). We were not able to analyze the expression level of *BRCA1* in ovarian cancer patients due to lack of RNA samples. However, we found extensive disorganization in the pattern and levels of methylation across the 23 CpG sites in the promoter region as compared to that in cancer-free woman and newborn carriers (Fig. 2i).

## Discussion and conclusions

In this study, we have screened a total of 865 females for their WBC *BRCA1* and *MGMT* promoters’ methylation status by the MSP assay. The overall frequencies were 8.7% for the *BRCA1* and 11.7% for the *MGMT* gene promoter. Remarkably, we found the frequency of *BRCA1* methylation to be similar in both newborns and adult females and are analogous to our previously reported frequencies [11, 24]. Importantly, both newborn and adult samples showed identical pattern and levels of methylation across all the studied CpG sites in the *BRCA1* promoter. This indicates that constitutional epimutation of the *BRCA1*gene is present from the early life of the carriers, as opposed to the belief that it is acquired later on during the lifetime of the individual.

The frequencies of *BRCA1* and *MGMT* methylations in delivering women were about 26% lower than that of both adult and newborn females, suggesting that the *BRCA1* and *MGMT* promoters are demethylated in women during pregnancy. Indeed, it has been reported that pregnancy reprograms the epigenome as a protective mechanism against breast cancer in women [25]. In addition, it was found that the IGF acid labile subunit, which is responsible for transporting the IGF1 protein in the blood circulation, is activated by hypomethylation whereas the IGF1R is silenced by hypermethylation [26]. Thus, the epigenetic modifications of these two genes could contribute to the protective outcome of early pregnancy and parity against breast cancers. Hence, it is plausible that in a portion of the delivering women, the *BRCA1* and *MGMT* promoters are demethylated due to either parity or early pregnancy as a protective mechanism against breast and ovarian cancers. However, further studies with larger sample size are needed to verify this.

Our group is the first to report the transmission of the *BRCA1* and *MGMT* epimutations from mothers to daughters. Although the overall frequency of inheritance was low, 1.4% for *BRCA1* and 3.1% for *MGMT*, it accounted for a high proportion of the mother carriers. In a recent report, the authors have concluded that *BRCA1* methylation is not transmitted from mother to daughter [27]. The discordance between the two studies could be due to the sample sizes, 6 mother-daughter pairs versus 290 pairs in our study. Although, in our study, *BRCA1* methylation was not transmitted from father to daughter, we cannot rule out the potential inheritance through paternal germ line as only one father was tested. However, we can conclude from this result that the majority of *BRCA1* epimutation appears to occur during early development, which could be due to an exposure to environmental insults. The finding that *BRCA1* mother carriers have *MGMT* newborn carriers, and vice versa may indicate a possible link between the constitutional epimutation of these two genes. Additionally important, it does rule out the possibility of contamination of maternal blood in cord samples.

The inheritance of methylated cancer-associated genes has been previously reported [21, 22]. As constitutive methylation of *BRCA1* and *MGMT* has been found to associate with an increased risk of cancer development [8–13, 28], it is conceivable to believe that the affected daughter has a high risk for developing these cancers. Indeed, it has been reported that a mother with constitutional *MLH1* and who had Lynch syndrome has transmitted *MLH1* epimutation to two of her children who developed also early colonic tumors [23].

It is still not clear whether epimutational inheritance occurs per se or it arises due to cross linkage to cis-acting genetic lesions. Several studies have revealed the constitutional epimutation of tumor suppressor genes to be linked to *cis*-acting genetic lesions [29–31]. As no such genetic lesion has been found in the promoter of *BRCA1* to explain its methylation [13], the inheritance of *BRCA1* methylation, we report in this study, may support the concept of transgenerational epigenetic inheritance.

In this study, we report a high frequency of constitutional *BRCA1*and *MGMT* methylation in breast and ovarian cancer. The detection of methylated *BRCA1*in WBC from ovarian cancer was reported previously in 20 out of 154 cases [32]. Although several studies have shown high frequencies of methylated *MGMT* promoter in breast and ovarian tumor tissues, our study is the first in finding the methylated *MGMT* in patients’ peripheral WBC [19, 20, 33, 34] suggesting that as in *BRCA1*, *MGMT* epigenetic modification in WBC also predispose women to breast and ovarian cancer. While we found a significant association between constitutional *BRCA1* methylation and early onset breast cancers (≤ 40 years) [11, 24], the constitutional *MGMT* methylation was significantly associated with late onset (≥ 50 years). Our results are in concordance with a previous study where a weak association was found between *MGMT* methylation with advanced age in triple negative breast cancers [19].

The analysis of the pattern and levels of methylation across the CpG sites in the *BRCA1* promoter region revealed that this pattern was very well-defined in the newborn and adult carriers but it was highly disorganized in the breast and ovarian cancer patients. Although in newborn carriers, we found high methylation levels in a region known to have strong promoter activity; this did not decrease the *BRCA1*expression. This is in accord with the argument that constitutional methylation is mono allelic [35]; consequently, only one allele of the *BRCA1* gene is methylated in the newly born carriers. However, according to the Knudson’s two-hit hypothesis, in the breast cancer patients, the two alleles are affected through the progress of the patient’s life [36]. Indeed, in the woman carriers, a twofold decrease in the expression level of the *BRCA1* mRNA was found in three out of five individuals, while the highest level of reduction in *BRCA1* expression was detected in breast cancer cases, which, interestingly, correlated highly (*R* = 0.89) with patient’s age reflecting the association between *BRCA1* promoter methylation and the early onset of the disease. Importantly, lower *BRCA1* expression was detected in blood leukocytes from healthy unaffected *BRCA1*mutation carriers as compared to that in controls [37] indicating the similarity between the effect of methylated and mutated *BRCA1*.

In conclusion, we have clearly shown:

1. The transmission of both *BRCA1* and *MGMT* epimutations from mother to daughter.
2. The frequencies of *BRCA1* and *MGMT* epimutations in female newborns are similar to that of cancer-free women.
3. Our data point at the possible demethylation of *BRCA1* and *MGMT* through reprograming of the epigenome during pregnancy.
4. *MGMT* epimutation is associated with ovarian cancer and the late onset of breast cancer.
  Our study sheds some light on the potential use of epimutations in cord blood as predictive biomarkers for cancer.

## Methods

### Study population

The study was approved by the Human Research Ethics Committee of the King Faisal Specialist Hospital and Research Centre according to the Declaration of Helsinki. All participants provided written consent before participation. Ten milliliters of cord blood and 10 ml of maternal peripheral blood were collected at the time of delivery at Al Yamamah Hospital (Riyadh), age of mothers range 19–46 years. Additionally, 10 ml of fresh peripheral blood was collected from cancer-free females, age range 15–50 years, and from breast and ovarian cancer patients coming to the oncology department in King Faisal Specialist Hospital and Research Centre in Riyadh, Saudi Arabia. Clinicopathological data (age, histological grade, and ER and PR status) were provided by the Department of Pathology. All blood samples were collected into EDTA tubes.

### Blood DNA and RNA isolation

Blood samples were immediately centrifuged at 2000×*g* for 10 min at 4 °C, and WBCs were carefully collected and transferred into two 2-ml Eppendorf tubes, one containing 900 ml RBC Lysis solution for subsequent DNA extraction using the Gentra Puregene Blood Kit and the other tube contained 1.2 ml RNALater solution for subsequent RNA extraction using RiboPure Blood Kit (Ambion).

### Methylation-specific PCR

DNA was treated with sodium bisulfate DNA and purified using EpiTect Bisulfite Kit (Qiagen) following the manufacturer’s recommendations. The DNA was then amplified using published PCR primers for *BRCA1* and *MGMT* [38, 39] that distinguish methylated and unmethylated DNA. PCR products were electrophoresed on 2% agarose gels and stained with Ethidium bromide. Totally methylated bisulfite-treated DNA was used as positive control. All the PCR reactions were repeated at least twice.

### Bisulfite pyrosequencing

DNA methylation was quantified by bisulfite pyrosequencing. Five different assays were designed using the PyroMark Assay Design software (Qiagen) in order to analyze the methylation status of 23 CpG sites across the *BRCA1* promoter. All the primers used in PCR amplifications and sequencings are listed in Table 4. The PCR and pyrosequencing reactions were performed using PyroMark products and reagents (Qiagen) as previously described [40]. Methylation quantification was performed using PyroMark Q24 software (Qiagen).

**Table 4:** Bisulfite pyrosequencing and real-time PCR primers

	Primers sequences	No. of CpG sites	Annealing temp
F1 R1Bio Sequencing	GGTATTGGATGTTTTTTTTTATAAGATTAT CCAATCCCCCACTCTTTC ATTATAGTTTTTAAGGAATATTGTG	3	56
F2 R2 Sequencing	GAAAGAGTGGGGGATTGGGATT AAAATACCTACCCTCTAACCTCTACT ACCTCTACTCTTCCA	4	60
F3 R3 Bio Sequencing	AGGGTAGGTATTTTATGGTAAATTTAGGT TATCTAAAAAACCCCACAACCTATCC ATGGTAAATTTAGGTAGAATTTT	5	60
F4 R4Bio Sequencing	AGATTGGGTGGTTAATTTAGAGT TCTAAAAAACCCCACAACCTATCC GGAAAAGAGAGGGAATTATAGATAA	6	58
F5 R5 Bio Sequencing	GGGGTAGATTGGGTGGTTAA TTATCTAAAAAACCCCACAACCTATC GAGAGGTTGTTGTTTAG	5	58
*BRCA1*	F 5′–TGTAGGCTCCTTTTGGTTATATCATTC–3′ R 5′–CATGCTGAAACT TCTCAACCAGAA–3′		59 °C
β- *Actin*	F 5′–TCC CTG GAG AAG AGC TAC GA–3′ R 5′–TGA AGG TAG TTT CGT GGA TGC–3′		59 °C

*F* forward, *R* reverse

### Real-time PCR

cDNA was generated from RNA by Superscript III (Invitrogen) reverse transcriptase and random hexomers. Quantitative real-time PCR was then performed with primer pairs specific for *BRCA1* transcript using *Actin* as an internal control. Primers are listed in Table 4. PCR was performed with SYBR green using CFX96 Real-Time System (Bio-Rad). The relative *BRCA1* expression was calculated based on the threshold cycle (Ct) value using the 2^−ΔΔct^ method. The fold change of mRNA expression was done relative to unmethylated cancer-free women for breast cancer patients and woman carriers and relative to unmethylated babies for the newly born baby carriers.

### Statistical analysis

General linear regression (GLM) was performed to determine the statistical significance for the association between *BRCA1*and *MGMT* promoter methylation and age of patients. All observed differences were considered to be significant when associated with a *p* value < 0.05.
